# Implementing alternative estimation methods to test the construct validity of Likert-scale instruments

**DOI:** 10.4069/kjwhn.2023.06.14.2

**Published:** 2023-06-30

**Authors:** Chang Gi Park

**Affiliations:** Department of Population Health Nursing Science, College of Nursing, University of Illinois Chicago, Chicago, IL, USA

## Introduction

A manuscript recently published in *Nursing Research* [1] suggested using polychoric correlations and polychoric confirmatory factor analysis (CFA) for unbiased assessments of construct validity in Likert-scale instruments, rather than Pearson correlations and Pearson correlation-based CFA. An editorial in the most recent issue of *Psychological Test Adoption and Development* also recommended the weighted least square mean and variance-adjusted (WLSMV) method for CFA-based validity testing [2]. Using polychoric correlation for CFA involves applying CFA estimation methods to ordinal item variables. However, relatively few nursing studies have used this estimation method to test the construct validity of ordinal variables.

As a general recommendation, the maximum likelihood (ML) method can be used for instruments with 5 to 7 item categories, as seen in the Likert scales commonly employed in nursing research [3]. The frequent application of strict cutoff rules for model fit indices to evaluate construct validity based on CFA estimation results may lead to an underestimation of the study instrument and modification of the CFA model by removing items or introducing connected item residual terms.

Therefore, better assessment methods of the construct validity of Likert scales are needed, and alternative estimation methods are recommended to avoid incorrect parameter estimates, such as factor loading coefficients, standard errors, and model fit statistics [4]. In this context, the purpose of this paper is to explain the necessity of alternative estimation methods and to present how those methods can be applied using affordable, accessible, and appropriate structural equation modeling (SEM) programs.

## Current practice for testing Likert-scale item validity

Construct validity testing for Likert-scale instruments has been conducted using the ML estimation method for CFA, with the assumptions of multivariate normality and an interval scale. For the Likert scale, ordinal item variables with 4 or 5 categories have commonly been treated as continuous variables, allowing the application of the ML estimation method. However, for 2 or 3 categories, alternative estimation methods other than ML must be applied [5]. At that time, the limited availability of software supporting alternative estimation methods posed a significant barrier, preventing nursing researchers from applying non-ML estimation methods for Likert-scale instrument evaluation using CFA [5]. Finney and DiStefano [6] recommended using ordinal CFA estimation methods such as WLSMV, regardless of the number of categories, if Mplus software (Muthén & Muthén, Los Angeles, CA, USA) was available. They also suggested employing the ML estimation method for Likert-scale variables with more than five categories. Additionally, the ML estimation method was recommended for five-category scales with a small, symmetrically distributed sample [3].

However, although the ML method has been recommended for the CFA model with five to seven categories, the estimation results may still exhibit biases [3,7]. For five categories, a downward bias of factor loading coefficients and associated standard errors were observed in a simulation study [7]. Furthermore, the ML method with five or more categories still demonstrated a relative 10% bias in estimated coefficients [3]. Similar biases were detected with additional categories; for example, ML estimation with a 7-point Likert scale still yielded biased estimates [8]. Thus, these studies support the use of non-ML methods for ordinal variables, regardless of the number of categories.

The application of ML for categorical variables can potentially yield inaccurate statistics, including standardized factor loading coefficients, standard errors, and global model fit statistics (e.g., the Tucker-Lewis index [TLI] or comparative fit index [CFI]) [9,10]. When the study sample size is small, the bias may be more severe. Consequently, for instrument revision, it is important to avoid unnecessary changes based solely on a single statistical criterion, as this may lead to a misleading evaluation of the instrument.

## The weighted least square mean and variance-adjusted estimation method for Likert-scale item validity testing

As the most highly recommended alternative CFA estimation method, the WLSMV estimation method is specifically designed for ordinal item data using Likert-scale instruments. This method provides more accurate statistics for construct validity testing than the ML-based estimation method [2]. The WLSMV estimation method for ordinal scale data was first introduced by Muthén et al. [11] and has since been used as a default method for models with categorical variables. The WLSMV is a robust version of diagonally weighted least squares (DWLS) and it provides valid estimates of adjusted fit statistics (Satterthwite, Satorra-Bentler, Scaled and Shifted or bootstrapped), and standard errors (robust and bootstrap). Another recommendation for Likert-scale item analysis is to apply the WLSMV method, regardless of whether the number of categories is <5 or ≥5, if Mplus software is available [6].

## Applications in nursing journals

A brief PubMed search for studies applying the WLSMV estimation method to validate Likert-scale instruments published in international nursing journals identified 13 papers. The WLSMV method was applied for the validity and reliability testing of the 6-Item State Anxiety Scale [12] and Self-Care of Heart Failure Index Score [13,14]. Since then, 10 more studies have been published [15-24]. These manuscripts used Mplus software to apply the WLSMV estimation method for the validity evaluation of Likert-scale instruments, most likely because nursing researchers had limited access to WLSMV-capable SEM software.

## Does the weighted least square mean and variance-adjusted method need more samples than maximum likelihood?

According to previous studies, the recommended sample sizes for WLSMV estimation are not significantly different from those for ML estimation. For instance, one study stated, “The sample size for the WLSMV estimate was not allowed to be larger than the sample size for the ML estimate.” [9]. Some studies have supported a sample size of over 200 for WLSMV [3,10], while others have recommended a sample size of 200 to 500 [25]. Based on this brief review of the required sample size for WLSMV, it appears that the recommended sample sizes are quite similar to the typical sample sizes for CFA using the ML estimation method. As a result, it is advisable to use WLSMV for construct validity tests if the study sample size is sufficient for the ML method.

## Structural equation modeling software for the weighted least square mean and variance-adjusted method

The Mplus program includes the WLSMV estimation method for ordinal data. The estimator option is defined as “ESTIMATOR=WLSMV,” which is contingent upon specifying “CATEGORICAL=ordinal variable name list.”

For nurse researchers who are unable to utilize Mplus due to financial constraints, the freely available R software with WLSMV estimation capability is now the ideal choice. The R package “lavaan” incorporates the WLSMV estimation method. The lavaan syntax for CFA, including the estimator option and the ordinal scale option, can be defined as follows:

cfa(..., estimator="WLSMV", ordered=TRUE)

When all variables are categorical, ordered=TRUE will automatically apply the WLSMV method without defining the estimator as WLSMV.

For those who do not use the R package or cannot afford commercial SEM software such as Mplus or Lisrel for CFA estimation, there are now two software programs, namely JASP and jamovi, that enable nurse researchers to run the R-based SEM package lavaan through a menu selection method similar to the SPSS menu-based interface. The JASP program can be downloaded from https://jasp-stats.org/. The current version of JASP is 0.17.2 and includes an SEM module capable of running the lavaan program. However, JASP only supports the DWLS estimation method, even though the original lavaan program also offers WLSMV as a robust DWLS estimation method. Due to this limitation, the JASP DWLS estimation method cannot provide robust DWLS results. Therefore, to utilize WLSMV estimation, the R lavaan program must be employed.

The latest version of the jamovi package now includes SEMLj, which offers the ability to utilize all CFA estimation method options available in the lavaan program. You can download the jamovi program from https://www.jamovi.org/. The current version is 2.3.21. The SEMLj module is an interface between jamovi and the R package lavaan [26]. Estimation method options for ordinal item scales are incorporated within the program. The “automatic” (default) option enables the lavaan program to choose the estimation method. However, it is essential to confirm the automatic selection of the estimation method for ordinal item variables. https://semlj.github.io/index.html presents examples and easy-to-follow instructions. Both lavaan CFA with the WLSMV option and jamovi SEMLj WLSMV yield the same estimation results as Mplus WLSMV. The ULSMV method, a lesser-known alternative, is also available in the lavaan program, and jamovi SEMLj can access this function as well.

A few critics have objected to the use of identical cutoff points for various estimation methods, as the current recommendations for these cutoff points were derived from a simulation study that employed the ML estimation method with multivariate normality assumptions [27-29]. However, only a few possible alternatives have been explored.

## Comparisons of the maximum likelihood and the weighted least square mean and variance-adjusted methods with a sample dataset

To illustrate the differences in CFA results estimated by ML and WLSMV methods, a manuscript with accessible raw data published in a nursing journal was chosen. The study aimed to assess the psychometric properties of the 24-item, 5-point Likert scale Arabic version of the Irish Assertiveness Scale among Saudi undergraduate nursing students and interns [30]. The initial four-factor CFA model with 23 items was estimated using the ML method. The authors noted that the fit indices, including root mean square error of approximation (RMSEA), CFI, TLI, and standardized root mean square residual (SRMR), were insufficiently satisfactory to accept. To improve the model fit statistics, a revised CFA model excluding three items was reestimated. However, the model fit indices of the revised model did not meet the minimum recommended cutoff points. The final model, which included four correlated item residual terms, reported CFI=0.89, TLI=0.86, RMSEA=0.06, and SRMR=0.08.

To compare the results of CFA differences using the WLSMV method, we accessed the study data provided online. This time, we estimated the CFA models with Mplus version 8.8 using both ML and WLSMV methods. The initial CFA model using the ML method displayed poor fit indices with RMSEA=0.065, CFI=0.833, TLI=0.811, and SRMR=0.064. However, the model fit statistics for the CFA model using WLSMV showed improvement with RMSEA=0.066, CFI=0.915, TLI=0.904, and SRMR=0.072. Since the model fit indices using WLSMV already met the recommended cutoff points, it might not be necessary to revise the CFA model solely due to poor model fit statistics. Nevertheless, the standardized factor loading coefficients of the three removed items were below 0.3. Based on the recommended cutoff point of 0.3, these three items could be removed.

For the CFA model with 20 items using the ML estimation method, the indices were RMSEA=0.071, CFI=0.849, TLI=0.825, and SRMR=0.06; however, with WLSMV, the indices were RMSEA=0.075, CFI=0.92, TLI=0.907, and SRMR=0.067. Since the model fit indices surpassed the commonly recommended cutoff points it may not be necessary to modify the CFA model with 20 items with correlated item errors.

As illustrated in this example, the CFA estimation method for the Likert scale is crucial for determining construct validity with greater accuracy. Employing the appropriate estimation method for construct validity tests can help avoid unnecessary instrument revisions and inaccurate validity test outcomes when the model fit statistics of CFA results do not surpass the recommended cutoff points.

## Conclusion and recommendations

Nurse researchers have commonly been advised to use the ML estimation method for Likert scale construct validity tests, under the assumption that treating the ordinal scale as an interval scale would not cause significant estimation issues. CFA results, including model fit indices, factor loading coefficients, instrument evaluations, and modifications, have been based on this practice. However, it has been suggested that alternative estimation methods, other than ML, should be considered for CFA estimation of ordinal scales, rather than solely relying on ML for Likert-scale assessments of nursing instruments. Despite the potential for underestimation of factor loading coefficients and standard errors, as well as model fit indices due to the use of the ML estimation method instead of the WLSMV method for ordinal scales, the lack of SEM software enabling the availability, accessibility, and adaptability of alternative estimation methods has severely limited the application of non-ML estimation methods in nursing research. These limitations could lead to undervalued nursing instruments and unnecessary modifications.

Construct validity testing of Likert-scale instruments is common in nursing research, and the previously indicated limitations of SEM software accessibility for nursing researchers should no longer hinder the application of the ordinal CFA WLSMV method, which is available in the R program. As presented in this manuscript, interface-based software, such as jamovi and JASP version 0.12.2 (JASP Team, 2020) now facilitate accurate evaluations of nursing instruments.

Understanding the different estimation methods, the availability of affordable software, and the appropriate use of these methods is important, since properly selecting an estimation method can avoid unnecessary instrument modifications to improve reliability and construct validity.

The choice of the CFA estimation method also influences the reliability test results for Likert-scale instruments. The composite reliability coefficient, an alternative to Cronbach’s alpha, has been recommended based on CFA estimation results. It is crucial to recognize that if the CFA estimation methods impact the estimated loading coefficient size and standard error, the recommended WLSMV estimation method for the Likert scale will also affect the estimated composite reliability coefficients. The WLSMV method was employed to assess the reliability of the 4-point ordinal scale Self-Care of Heart Failure Index Score using CFA [13,14]. The ordinal reliability coefficient, which utilizes polychoric correlations, should be considered an essential reliability test method for nursing researchers [31].

Currently, SEM software offering alternative estimation methods for the Likert scale is available and even freely accessible to nursing researchers. Utilizing these available estimation methods can enhance psychometric evaluation in nursing research. Moreover, the application of alternative estimation methods has the potential to enhance the quality of instrument development.
